# Testing for Local Adaptation to Spawning Habitat in Sympatric Subpopulations of Pike by Reciprocal Translocation of Embryos

**DOI:** 10.1371/journal.pone.0154488

**Published:** 2016-05-03

**Authors:** Hanna Berggren, Oscar Nordahl, Petter Tibblin, Per Larsson, Anders Forsman

**Affiliations:** Centre for Ecology and Evolution in Microbial Model systems, EEMiS, Department of Biology and Environmental Science, Linnaeus University, SE-391 82 Kalmar, Sweden; University of Connecticut, UNITED STATES

## Abstract

We tested for local adaption in early life-history traits by performing a reciprocal translocation experiment with approximately 2,500 embryos of pike (*Esox lucius*) divided in paired split-family batches. The experiment indicated local adaptation in one of the two subpopulations manifested as enhanced hatching success of eggs in the native habitat, both when compared to siblings transferred to a non-native habitat, and when compared to immigrant genotypes from the other subpopulation. Gene-by-environment effects on viability of eggs and larvae were evident in both subpopulations, showing that there existed genetic variation allowing for evolutionary responses to divergent selection, and indicating a capacity for plastic responses to environmental change. Next, we tested for differences in female life-history traits. Results uncovered that females from one population invested more resources into reproduction and also produced more (but smaller) eggs in relation to their body size compared to females from the other population. We suggest that these females have adjusted their reproductive strategies as a counter-adaptation because a high amount of sedimentation on the eggs in that subpopulations spawning habitat might benefit smaller eggs. Collectively, our findings point to adaptive divergence among sympatric subpopulations that are physically separated only for a short period during reproduction and early development—which is rare. These results illustrate how combinations of translocation experiments and field studies of life-history traits might infer about local adaptation and evolutionary divergence among populations. Local adaptations in subdivided populations are important to consider in management and conservation of biodiversity, because they may otherwise be negatively affected by harvesting, supplementation, and reintroduction efforts targeted at endangered populations.

## Introduction

Local adaptation is typically studied among populations that are allopatric [1, 2]. Hence, there exists considerably fewer examples of local adaptations among sympatric populations (but see Richter-Boix *et al*. [3], Fraser and Bernatchez [4]). Within fine spatial scales, high connectivity among adjacent areas may facilitate gene flow, thus potentially preventing genetic subdivision and evolution of local adaptations—especially in aquatic ecosystems where organisms are not normally as limited by dispersal boundaries [5].

As put forth by Williams [6]; an adaptation is a phenotypic feature which is a product of past natural selection. If environmental conditions vary among locations and influence survival of early life stages, such as eggs or larvae, this may lead to local adaptation in early life-history traits [7] or reproductive strategies [8]. Such strategies may manifest as variation among populations in, for example, reproductive effort, egg size, growth rate, and size at maturity [8–12]. For instance, females may either produce many eggs of lesser quality, or fewer eggs of higher quality [13, 14]. However, empirical support for the occurrence of local adaptations in the aforementioned traits among sympatric populations is rare.

Adaptations to local environment could give individuals higher relative fitness in their native habitat compared to alternative habitats [2], and also higher relative fitness in their local environment than immigrant individuals. However, few traits are strictly genetically influenced [15–17]. Phenotypic variation among individuals and populations may represent underlying genetic differences, be a consequence of environmentally induced phenotypic plasticity, or reflect a combination of the two [17–22]. The range of phenotypes that can be produced by a given genotype within a population in combination with different extrinsic and intrinsic factors can be described by a reaction norm [15, 16]. The shape of the reaction norm may vary among genotypes (*i*.*e*. genotype by environment interactions), and this can contribute to phenotypic differences both within and among populations [23, 24]. High levels of phenotypic and genetic variation help populations to withstand environmental changes and can facilitate evolutionary modifications [25–28]. Knowledge of population genetic structures and local adaptations is a key to developing successful management strategies for harvested and protected populations [29, 30].

One classical approach to experimentally test for local adaptation is to perform reciprocal translocation experiments [2, 31, 32]. Such experiments can also be used to evaluate whether there exists genetic variation and gene-by-environment interactions within populations [23].

In this study, we tested for evolutionary divergence and local adaptation in traits associated with reproduction in two sympatric subpopulations of anadromous pike (*Esox lucius*) on the southeast coast of Sweden in the Baltic Sea. *Esox lucius* is a long-lived, iteroparous, large keystone predator fish species that is an emerging model organism for studies in ecology and evolutionary biology [33]. Baltic Sea pike spawn for the first time at an age of 1–4 years and then normally on a yearly basis. Previous studies in our study system suggest that the majority of individuals mature at age 3–4 [34]. *Esox lucius* is widespread in the northern hemisphere [35, 36] and inhabits both fresh and brackish water environments.

**Fig 1:**
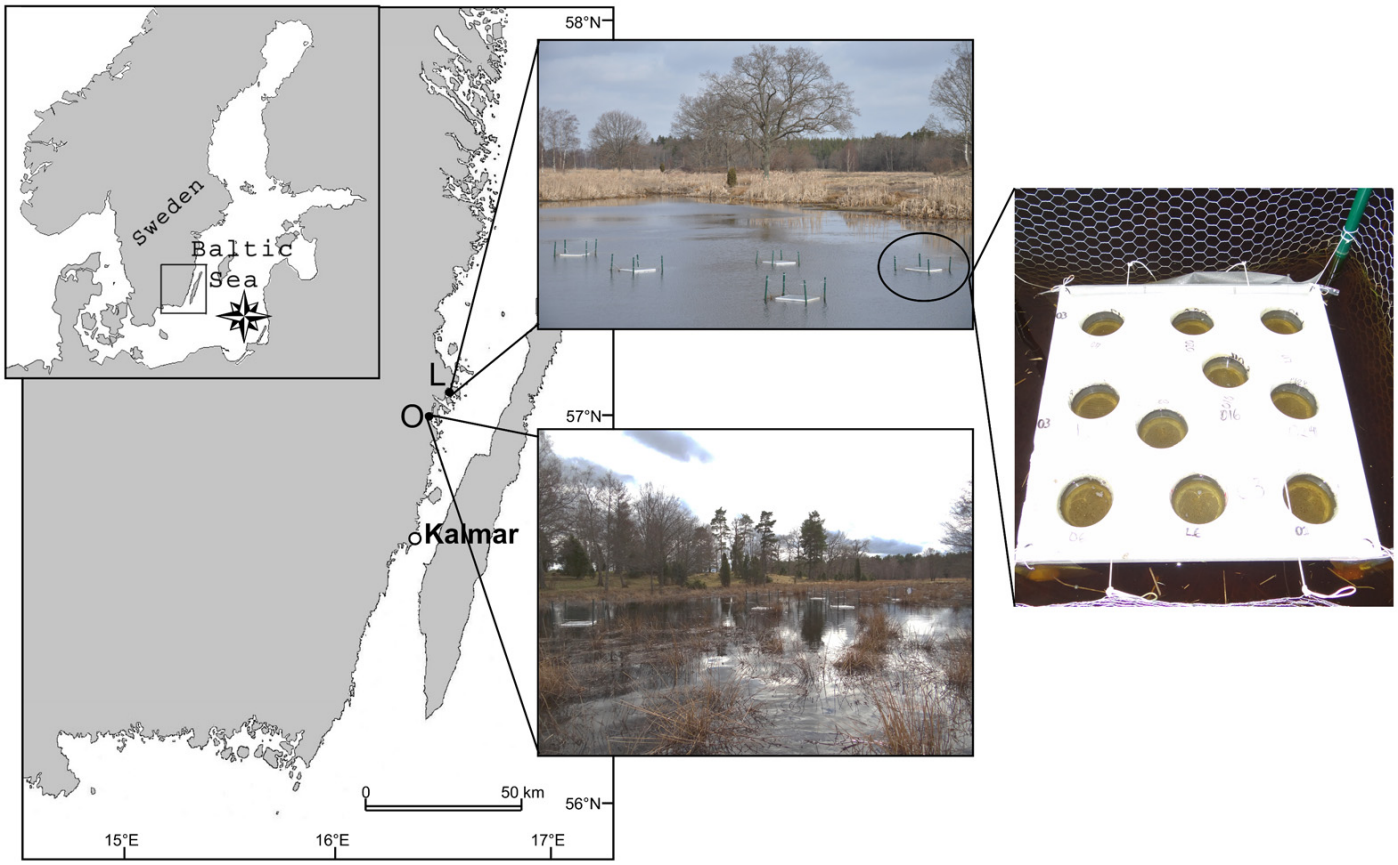
Map of study area in the Baltic Sea, southeast coast of Sweden, with marked spawning locations of the two studied populations of *Esox lucius*, Lervik (L) and Okne (O). The sites are separated by approximately 10 km. The map was reprinted from [34] under a CC BY license, with permission from [The University of Chicago Press], original copyright [2015]. Photographs show the different wetlands with the five experimental enclosures containing one raft each, and a single raft with holes for ten batches.

Our model system consists of genetically distinct subpopulations that spawn in different freshwater wetlands separated by a short geographic distance (~10 km, see Fig 1) relative to the dispersal capacity of *E*. *lucius* [34, 37]. Individuals spend only a few weeks in the freshwater spawning habitats during the reproductive and larval periods; hence, subpopulations are only physically separated for a fraction of the life cycle [37–39]. Outside the reproductive period, individuals are mixed and share a sympatric foraging habitat in the coastal areas of the Baltic Sea [34]. This study system thus offers good opportunities to study divergence and local adaptation in sympatric subpopulations that only encounter different environmental conditions and divergent selection for a short period [34, 37]. Local adaptations of sub-populations to spawning habitats in this system has previously been demonstrated with regards to divergence in growth of early life stages [34], and number of vertebrae [40].

We evaluated two assumptions regarding local adaptation to spawning environments by performing a reciprocal translocation experiment that included approximately 2500 *E*. *lucius* embryos in the wild. First, we tested the hypothesis that offspring should perform better at “home” than “away” [2] by investigating whether hatching success and survival was higher in the population’s native spawning habitat than in a foreign spawning habitat. Second, we tested the hypothesis that native population offspring should outperform non-native population offspring [31] by examining whether hatching success and survival was higher in the populations native spawning habitat compared to that of immigrants. Data from the reciprocal translocation experiment were also used to test for genotype by environment interactions [23] and to evaluate whether there was genetic variation within subpopulations upon which natural selection could act. Finally, we tested for differences in female reproductive effort (measured as the gonad mass in relation to somatic body mass) and size of eggs (measured as dry egg mass) between subpopulations.

## Materials and Methods

### Ethics

All applicable national guidelines for the care and use of animals were followed. Ethical approval for the study was granted by the Ethical Committee on Animal Research in Linköping, Sweden (approval 9–06 & 39–10). Permission for field studies was granted by County Administrative Board in Kalmar (approval 623-1681-13).

### Study area

*Esox lucius* used in this study originated from two subpopulations that spawned in different wetlands supplied by water from small streams, Lerviksbäcken and Oknebäcken (width ranging between 1–3 m, mean annual water flow of less than 0.5 m^3^/s). Both streams flowed through similar woodlands and agriculture areas, and had outlets located approximately 10 km apart on the southeast Baltic coast of Sweden [39] (Fig 1). The biotic and abiotic ecological conditions in the two wetlands differed. In Okne, the wetland consisted of flooded grassland with much submersed vegetation and hard-bottom. In Lervik, the wetland had soft-bottom, less submersed vegetation and higher amounts of suspended material in the water phase. During spring, between 500–1500 *E*. *lucius* migrate upstream from the Baltic to the actual spawning site in the wetlands [37] and reproduction predominately occurs over submerged vegetation [39, 41]. Natal homing behaviour has been inferred based on comparisons of elemental fingerprinting in the otoliths [42], and spawning migration and site fidelity established based on recapture histories of marked individuals [37, 43]. Analysis of microsatellite genetic markers confirm that gene flow between these two subpopulations is restricted (*pairwise F*_*ST*_ = 0.0668, *P* < 0.001) [34, 37].

### Reciprocal translocation experiment

Embryos used in the experiment were produced by artificial fertilization in the field. From each subpopulation, we made up parental pairs consisting of one male and one female (Lervik = 22 pairs, Okne = 18 pairs). We then employed a split-brood experimental design where half of the offspring was planted in the native habitat and the other half transplanted to the non-native habitat. We used only one male and one female for each split-brood, and each parental individual was only used for one split-brood.

During six days (between 15-Apr-2013 and 22-Apr-2013) *E*. *lucius* from both subpopulations were captured with fyke nets placed in the streams, thus caught during the migration to their own choice of spawning ground. Individuals were stripped of gametes into sterile tubes (50 ml falcon tubes for eggs and 2 ml Eppendorf tubes for milt). To ensure high quality of gametes, and because eggs may have been exposed to water if they lay close to the urogenital opening, we discarded the first batch from each ovulating female. Gametes were kept on ice (up to 2h) until fertilization. Without getting harmed, all *E*. *lucius* were safely returned to their natural habitats immediately after handling, to be able to spawn naturally as well.

Fertilization and subsequent distribution of batches to experimental set-ups were performed simultaneously in each wetland by two teams of researchers. This was to safeguard against results being influenced by different handling time. Eggs were counted (approximately 30 eggs per batch yielding a total of 2466 eggs divided amongst 40 families) and then mixed with milt (0.2 ml) for 10 seconds in a small glass dish (ø = 5cm). Thereafter, water from the current spawning habitat was added and the eggs were left for two minutes to promote egg hardening. The fertilization begins immediately when the egg gets in contact with water and opens up for the sperm [35, 44]. After two minutes the fertilized eggs were rinsed two times with water and then placed in separate plastic bins (height = 99 mm, ø = 99–116 mm from bottom to top) in which the bottoms had been replaced by a net (made of plastic with a mesh size of 1.5x1.5 mm) to allow water from the wetland to circulate in and out. The split-brood batches were randomly distributed among five different enclosures in each of the two wetlands (Fig 1). Each enclosure contained a 50x50x5 cm squared raft made of floating polythene with closed cells (LD45 –Recticel, Mönsterås, Sweden). Each raft had 10 holes in which the plastic bins with the fertilized eggs were placed.

### Quantification of hatching success and viability of larvae

All batches were examined and photographed (Sony Cybershot DSC-HXI100V digital camera, focal length 4.8–144 mm) every second to every third day. Hatching of 70 batches occurred approximately 13 days post fertilization (mean = 13.1, range = 11–16 days) We counted how many of the eggs that hatched (hatching success) and how many of the hatched larvae that survived through the yolk sac phase, resulting in binary data that also could be displayed as proportions. Experiment ended by an overdose of benzocaine at the end of the yolk sac phase (6–7 days post hatching).

### Evaluating female reproductive effort and egg size

To test for differences between subpopulations in reproductive investment strategies we sampled and sacrificed ripe but not ovulating females (*n* per subpopulation = 23), when animals were collected for the translocation experiment. Females with a total length up to 90 cm were used to achieve a sample of similar sized individuals from both subpopulations. Gonad dry mass, adjusted for body size, was used as an indicator of reproductive effort, and total length served as body size measurement. Female total length was measured to the closest millimetre using a measuring board prior to a careful dissection during which gonads were removed.

Egg dry mass was used as a measure of egg size and quality (in terms of nutrient content) [45]. Subsamples of ovarian tissue weighing 1 g (0.001 g accuracy, Milligram Balance PB303-s, Mettler-Toledo) were sampled from each of the anterior, middle and posterior parts of both ovaries, resulting in six subsamples per individual. The mean value computed across the six subsamples was used for the analyses of egg size. The numbers of yolked eggs in each subsample were counted, and egg mass was obtained by converting number of eggs per unit mass to dry mass per egg. Dry mass of gonads were obtained after drying to a constant mass at 50°C.

### Statistical analysis

We treated eggs as individual data points, rather than using family means for each batch, and applied Generalized linear models (GLMs) and Generalized linear mixed models (GLMMs) to binary data on hatching success and survival through yolk sac phase.

To evaluate whether subpopulations responded differently to translocation we tested for an effect of the interaction between treatment and origin by using GLMs with a binomial fit and a logit-link power function. Then we moved on to analyse populations separately to evaluate effects of treatment on hatching success and survival through yolk sac phase, respectively, in separate analyses. In these analyses we also evaluated whether the response to translocation varied among families by testing for an interaction effect of genotype (family) and environment on both hatching success and on survival through yolk sac phase. For these analyses we applied GLMMs with binomial fit where treatment was set as fixed effect, and family and the interaction between family and treatment were set as random effects. Statistical significance of the random factors was assessed using the log-likelihood ratio test with one degree of freedom per random effect [46]. Statistical significance of the fixed effects was assessed using the Wald z test.

Next, we tested for effects of translocation treatment between subpopulations (within spawning habitats) by using Generalized linear models (GLMs) with a binomial fit and a logit-link power function.

Statistical analyses were performed in the statistical software R Studio (Version 0.99.879–2009–2016 RStudio, Inc) using the lme4 package.

To test for differences between subpopulations in reproductive effort and egg size we used full factorial ANCOVA models. Each response variable was analysed separately, with IBM SPSS statistics 20.

## Results

### Effects of translocation on hatching success and survival through yolk sac phase

**Fig 2:**
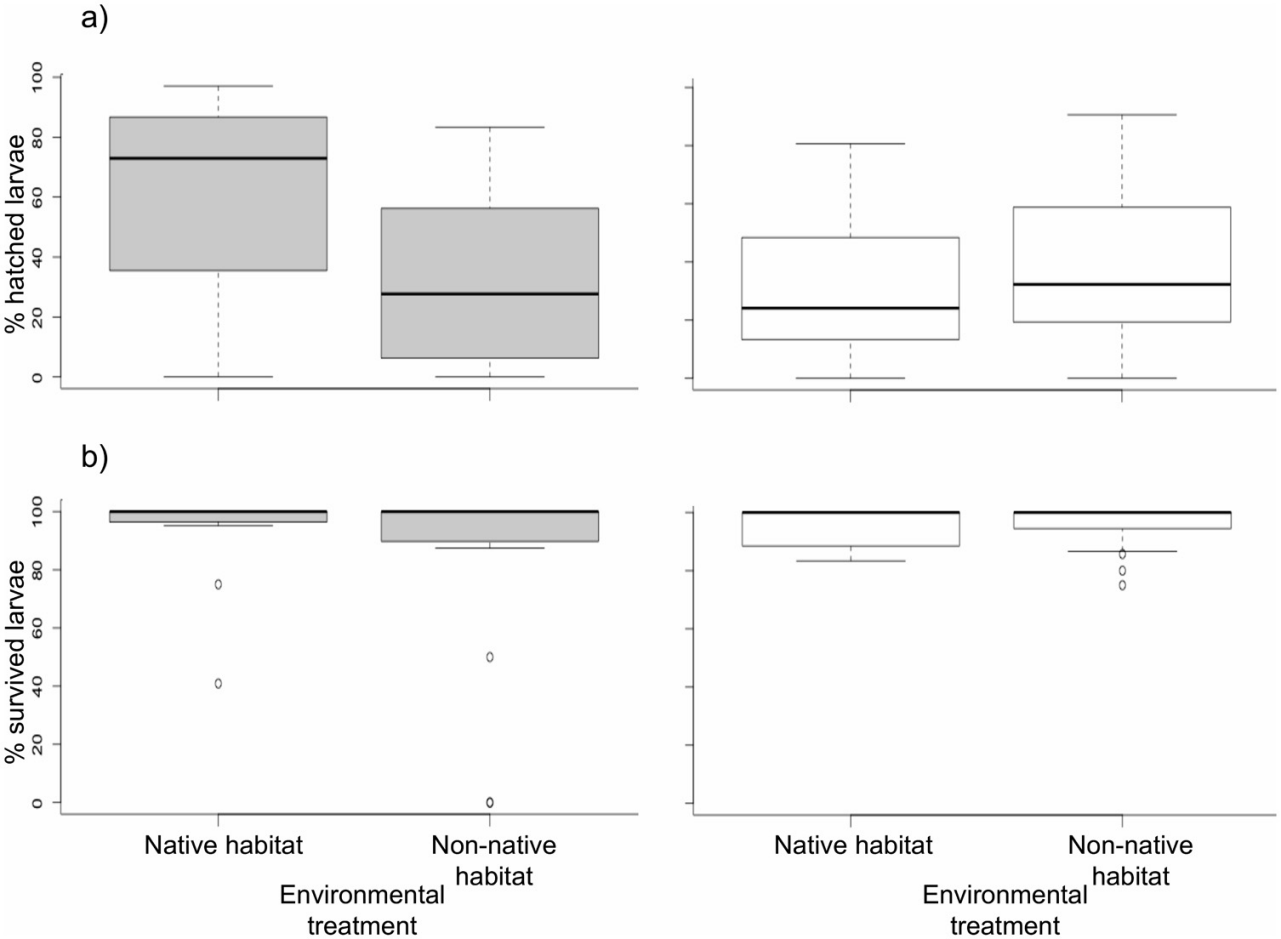
Within- and between-population comparisons of a) hatching success (%) and b) survival through yolk sac phase (%) for *E*. *lucius* reared in native and non-native environments using a reciprocal translocation experiment. The boxes are indicated with habitat treatment (native vs non-native) and population origin with grey colour for Okne and white colour for Lervik. The solid lines within the boxes indicate medians, the boundaries of the box indicate 25th and 75th percentiles, whiskers below and above indicate 10th and 90th percentiles, and dots indicate outlying observations.

For hatching success, subpopulations responded differently to translocation treatment (Generalized linear model, effect of treatment: *z* = 8.51, *P* < 0.0001; effect of population: *z* = 8.73, *P* < 0.0001, effect of interaction; *z* = 7.83, *P* < 0.0001) (Fig 2a). For survival through yolk sac phase, subpopulations did not respond differently to translocation treatment (GLM, effect of treatment: *z* = 0.72, *P* = 0.47; effect of population: *z* = 0.76, *P* = 0.45; effect of interaction: *z* = -0.45, *P* = 0.65) (Fig 2b). Families from Okne had higher hatching success in their native habitat compared to their non-native habitat (Generalized linear mixed model, effect of treatment: *z* = 3.81, *P* < 0.0001) (Fig 2a). No such difference between spawning habitats was detected amongst families from Lervik (*z* = 1.34, *P* = 0.18) (Fig 2a).

When testing for differences between populations in the Okne wetland, native families had higher hatching success compared to translocated non-native families from Lervik (GLM, effect of population: *z* = 6.64, *P* < 0.0001) (Fig 2a). In Lervik, there was no difference in hatching success between native families from Lervik and non-native families from Okne (*z* = 0.16, *P* = 0.87) (Fig 2a). Survival through yolk sac phase did not differ between native and non-native families neither within the spawning habitat Okne (GLM, effect of population: *z* = 0.82, *P* = 0.42) nor within the spawning habitat Lervik (*z* = 1.3, *P* = 0.19) (Fig 2b).

### Effects of family and genotype by environment interactions

**Fig 3:**
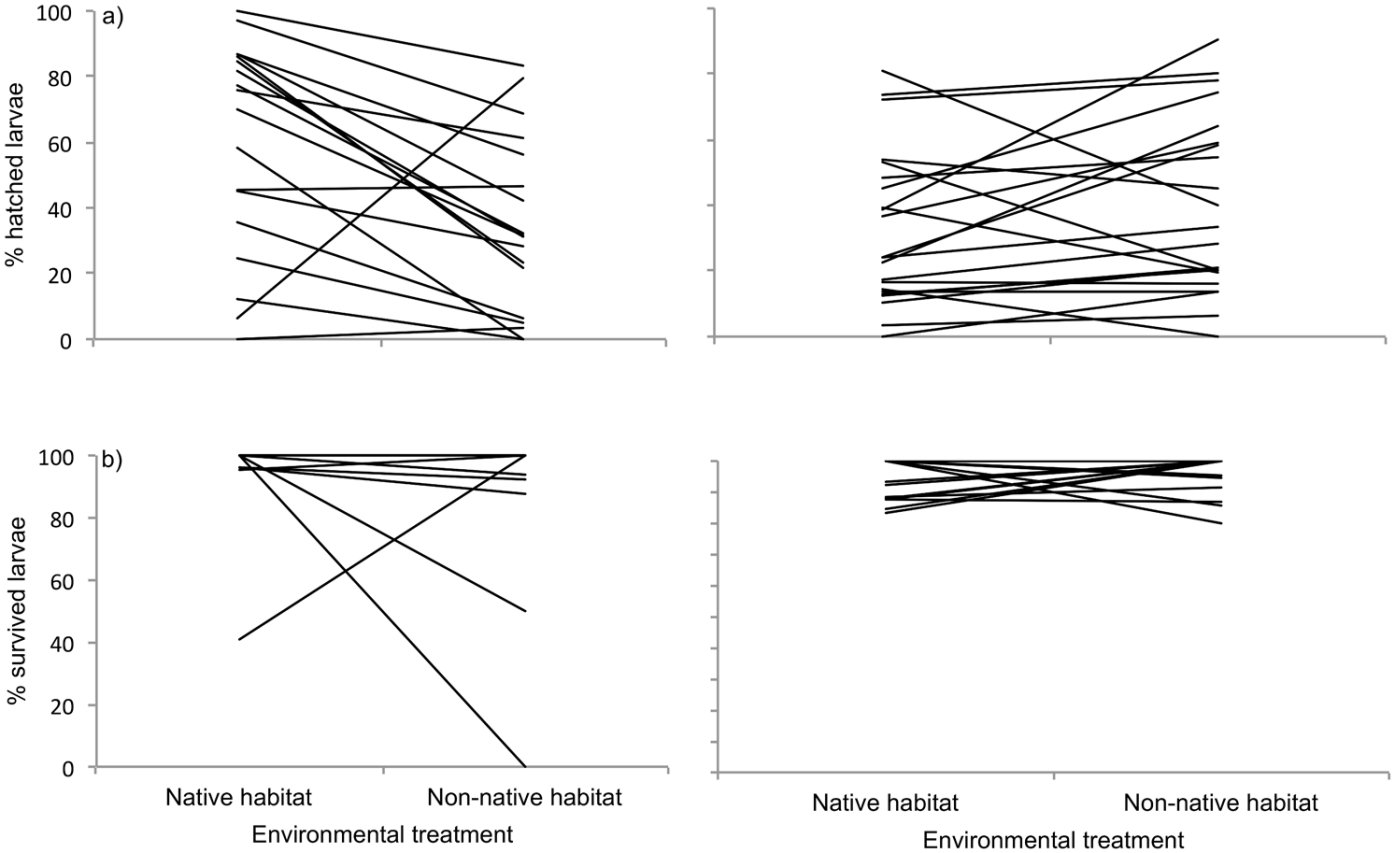
Genotype by environment interactions as indicated by pairwise within-family comparisons of a) hatching success (%), and b) survival through yolk sac phase (%) of *Esox lucius* depending on habitat translocation treatment (native vs non-native). Figure show results for Okne to the left and Lervik to the right. After removal of the outlier families showing the largest differences between treatments (one outlier for each of the Okne plots), the effect of translocation treatment still depended on family both for hatching success (χ^2^ = 1068, *df* = 1, *P* = 0.006) and survival through yolk sac phase (χ^2^ = 74.73 *df* = 1, *P* < 0.0001) in the Okne population.

The effect of translocation treatment on hatching success depended on family both in the Okne subpopulation (GLMM, effect of family by environment interaction: χ^2^ = 74.45, *df* = 1, *P* < 0.0001) and in the Lervik subpopulation (χ^2^ = 26.84, *df* = 1, *P* < 0.0001) (Fig 3a). The effect of translocation on survival through yolk sac phase also depended on family in Okne (family by environment interaction: χ^2^ = 19.05, *df* = 1, *P* < 0.0001), but no such interaction effect was apparent in Lervik (χ^2^ = 0.61, *df* = 1, 0.1 < *P* < 0.5) (Fig 3b).

### Reproductive investment strategies differed between subpopulations

**Fig 4:**
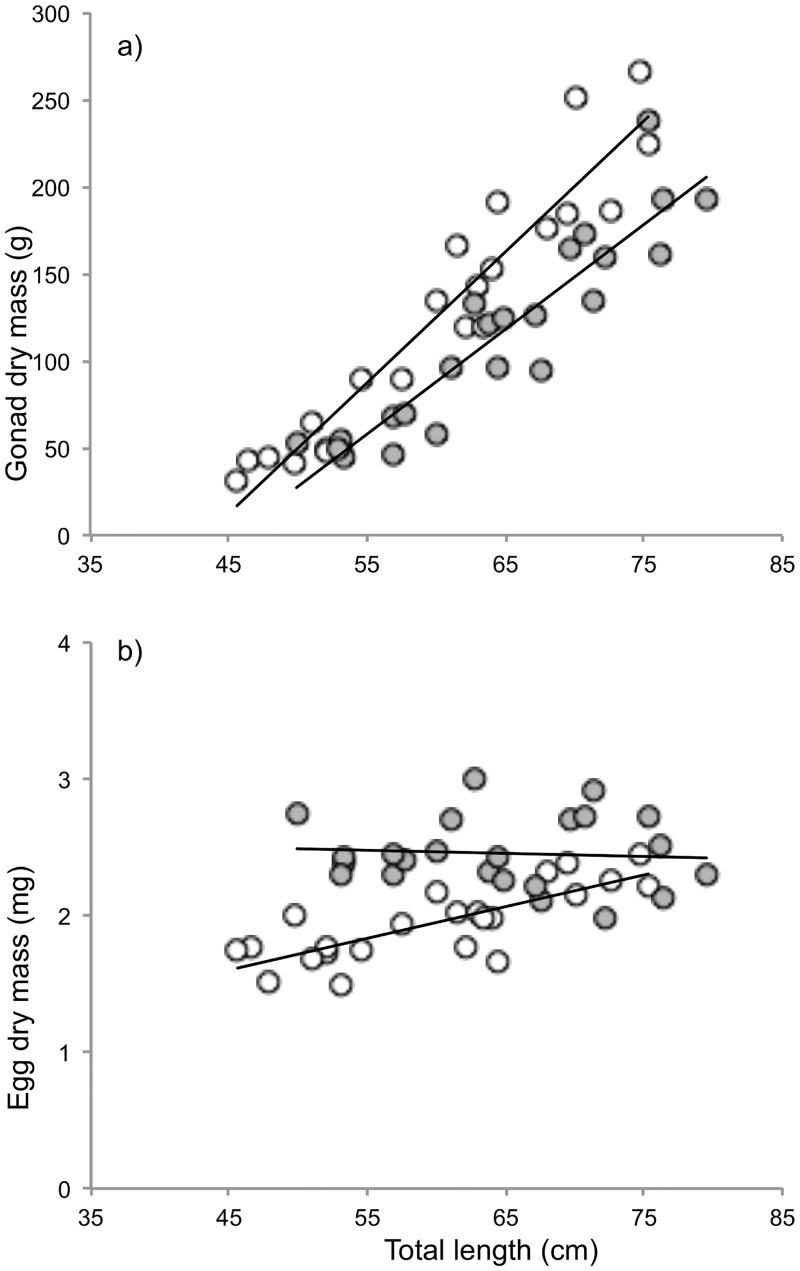
Between population comparisons of *Esox lucius* female reproductive allocation strategies. **a)** reproductive effort measured as gonad dry mass as a function of female body size, and **b)** egg size measured as dry mass per egg as a function of female body size. Every dot represents one individual and the same individuals are represented in both graphs. Grey dots indicate individuals from Okne (n = 23) and open circles indicate individuals from Lervik (n = 23).

Females from the two subpopulations invested different amounts of energy into their reproductive tissue in relation to their body size (ANCOVA, effect of population: *F*_1, 43_ = 32.7, *P* < 0.001; effect of body size: *F*_1, 43_ = 299.7, *P* < 0.001; effect of interaction: *F*_1, 42_ = 3.9, *P* = 0.055). Females from Lervik had a higher reproductive investment (140.3 g, SE = 4.92) than females from Okne (99.83 g, SE = 4.92) when compared at estimated marginal mean body length of 62 cm (Fig 4a).

Females allocated their reproductive effort between number and size of eggs differently depending on subpopulation and body size (ANCOVA, effect of population: *F*_1, 44_ = 18.43, *P* < 0.001; effect of body size: *F*_1, 44_ = 7.32, *P* = 0.01; effect of interaction: *F*_1, 44_ = 11.45, *P* = 0.002). Egg size increased with increasing female body size in Lervik but not in Okne (Fig 4b).

## Discussion

We report on differences in life-history traits between two sympatric subpopulations of anadromous pike (*E*. *lucius*) that spawn in separate wetlands but share a common forage habitat in the Baltic Sea outside the reproductive period. A reciprocal translocation experiment pointed to genetic variation and genotype by environment interaction effects on egg hatching success and larval viability within populations. The results from the translocation experiment further indicated local adaptation in one of the two subpopulations, manifested as enhanced hatching success of eggs (but not survival of yolk sac larvae) in the native habitat, both when compared to siblings transferred to a non-native habitat and when compared to immigrant genotypes from the other subpopulation. Moreover, data for adult females revealed differences between subpopulations in reproductive effort and egg size. We suggest that the observed differences in hatching success and reproductive strategies represented responses to abiotic and biotic conditions in the local spawning environments, and that responses manifested in different life-history stages in the different populations.

### Genotype by environment interaction effects on offspring viability

Local adaptations can only evolve if there is genetic variation for natural selection to act upon. Our translocation experiment demonstrated genetically based variation in traits associated with offspring viability, and that variation was present among families within subpopulations as well as between subpopulations. Non-genetic maternal effects may have contributed to the variation seen among families and subpopulations [47, 48], but the crossing norms of reaction (Fig 3) demonstrated that genetic components also were involved.

There were genotype by environment interaction effects on hatching success in both subpopulations. For survival through yolk sac phase, there was an interaction effect only in the Okne subpopulation. These findings showed that some families suffered from decreased offspring viability and some families benefitted from increased offspring viability after being translocated to a non-native spawning habitat. Such variation in reaction norms is necessary for the evolution of phenotypic plasticity [20, 49, 50] and could help subpopulations to better withstand environmental changes [17, 21, 27]. This is important for *E*. *lucius* (and other species) that use spawning habitats with large spatial and temporal variation in environmental factors.

### Subpopulations responded differently to reciprocal translocation experiment

Theory posits that locally adapted subpopulations have higher fitness in their native habitat than in alternative habitats, and predicts that there should be a negative effect of translocation to a foreign habitat [2, 31]. Such a negative effect was evident for hatching success in the subpopulation that naturally spawned in Okne (Fig 2a), suggesting that this subpopulation was adapted to the local spawning habitat. However, there was no effect of translocation on survival through yolk sac phase in neither of the studied subpopulations. Theory also posits that native individuals in locally adapted subpopulations should have higher relative fitness than immigrant individuals from foreign subpopulations [2, 31]. This prediction was fulfilled in the Okne subpopulation; eggs in these families had higher hatching success in their native habitat (Okne) compared with non-native eggs originating from the Lervik subpopulation (Fig 3a). For survival through yolk sac phase, there were no differences depending on population origin within the different spawning habitats. Egg and larval phases are known periods of high mortality in many fish species [51, 52]. That we found no effect of translocation on larvae viability in neither of the subpopulations suggests that the embryonic phase of *E*. *lucius* was more sensitive to extrinsic mortality factors than the first post-hatch period when living on the yolk.

### Female reproductive allocation strategies differed between populations

The reciprocal translocation experiment revealed signs of local adaptation in one of the subpopulations, but the processes behind adaptive divergence might have been operating in both [31]. Local adaptations related to early life stages can impact on life-history traits expressed later in life, for example parental reproductive investment strategies [8]. If mortality is high during early life stages, any adaptations that reduce mortality may have profound fitness advantages. Our data from the field study uncovered differences between subpopulations in reproductive effort and in egg dry mass. Egg size can be used as an indicator of egg quality in the form of nutrient content and furthermore, it has been shown that larger egg size is associated with increased larval survival—once hatched [45]. Females from Lervik invested more resources into reproduction and also produced more eggs in relation to their body size compared to females from Okne (Fig 4a). Females from Okne invested less in gonads in total, but allocated more energy into each egg compared to females from Lervik (Fig 4b). Changes in early life stages can impact on life-history traits expressed later in life, for example parental reproductive investment strategies [8]. In accordance with this notion, we suggest that females from subpopulation Lervik may have adjusted their reproductive strategies as a response to less favourable abiotic conditions in the spawning habitat, to increase the number of recruiting offspring and thereby spreading their chances (*i*.*e*. bet-hedging). We discuss this possibility in more detail below.

### What environmental factors might have contributed to the divergence between populations in different life-history traits?

**Fig 5:**
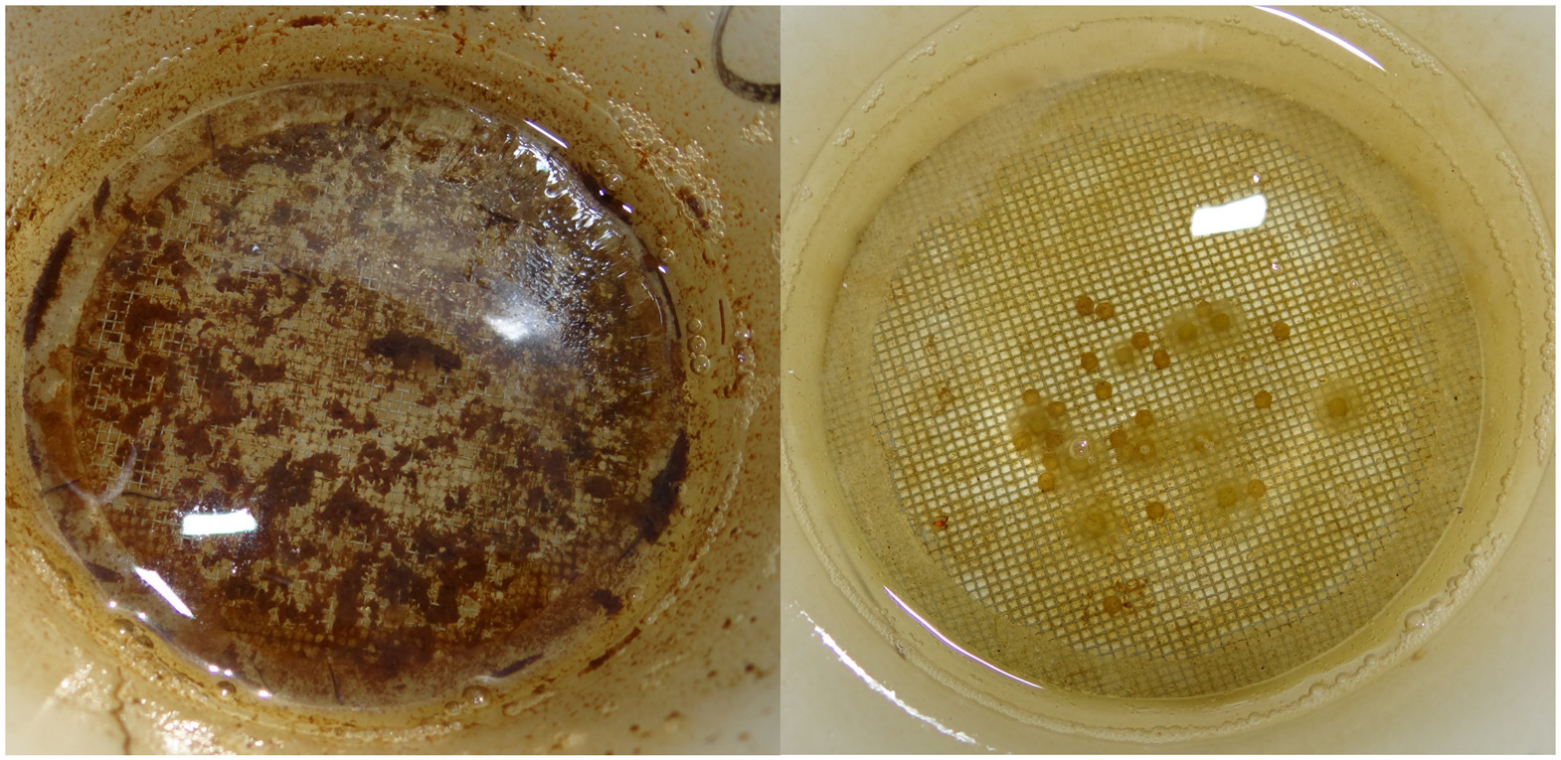
The two spawning wetlands differed in bottom substrate, causing sedimentation and particles that adhered to the surfaces of the *Esox lucius* eggs in Lervik. The pictures show egg batches from the same family, hence planted at the same time but in different wetlands: the batch to the left in Okne and the batch to the right in Lervik. This split-brood is two days old.

The Lervik wetland was less vegetated, had a different bottom substrate [39], and higher amounts of suspended materials in the water phase. This caused considerable sedimentation onto the eggs (Fig 5). Sedimentation of finer particles on eggs reduces hatching success in *E*. *lucius* [35, 44, 53] and other anadromous fish species [54]. This might explain the lowered hatching success for Okne offspring in the Lervik wetland. However, if the observed differences are due to habitat quality alone, why did Okne offspring outperform Lervik offspring in the Okne wetland? One reason for the observed difference in hatching success could be that females from Lervik produced smaller eggs. This reasoning builds on the assumption that egg size is positively correlated with hatching success and survival of larvae [55]. However, Murry *et al*. [45] found a negative correlation between egg size and hatching success in pike. An explanation based on lower hatching success of small compared with larger eggs also is inconsistent with the finding that hatching success was similar for both subpopulations in the Lervik wetland (Fig 2). We propose another explanation: that higher fecundity and smaller eggs produced by females from Lervik represent counter-adaptations or physiological adjustments caused by a feed-back loop from local abiotic conditions. In Lervik, smaller eggs may permit sufficient oxygen transfer and promote embryonic development and hatching success despite sedimentation [56]. It is thus possible that lower hatching success caused by sedimentation in Lervik has selected for increased fecundity in females originating from this population (Fig 4) [8, 13]. Conversely, females from Okne may produce fewer but larger eggs to increase survival probability of their offspring, under the assumption that biotic interactions such as competition and cannibalism were more intense in this environment compared with Lervik. This suggestion is based on the observation that when the numbers of emigrating fry were quantified in 2008, the number in Okne exceeded that in Lervik by >80000 whilst the number of spawning adults was similar in the two wetlands [34, 37, 39, 42].

Based on the present study approach we cannot determine whether the differences in reproductive allocation strategies between subpopulations were of genetic origin. However, for differences to represent phenotypic plasticity, adult females from the two subpopulations would probably need to utilize different foraging habitats. This seems not to be the case. Past investigations show that the subpopulations of *E*. *lucius* used in this study mix in the coastal waters outside of the reproductive period and thus share a common foraging habitat, utilizing similar resources [34]. The sympatric foraging area can be considered as an homogeneous ecosystem based on community analyses of macrophytes, invertebrates and fish showing very high similarities across sampling sites [34]. It is therefore plausible that the observed differences in reproductive strategies between females from Lervik and Okne reflected differences either in their ability to acquire resources or in the way they allocated resources between competing demands [57], not that they utilized different environments.

We cannot identify with certainty what extrinsic selective factor(s) might have contributed to the differences seen in our study system. Predation regimes and intensity of competition can shape the evolution of life-history strategies [10, 58–60]. However, it is unlikely that any of these factors was responsible for the variation in hatching success seen in our study. Differences in the intensity of competition for food, or cannibalism, also cannot account for the observed variation among families in survival of hatched larvae, because the experiment was terminated at the end of the yolk sac phase before larvae started to feed. Because performance of eggs and larvae was monitored in cages, predation can be excluded from the list of potential sources of variation in the experiment. Although predation and competition cannot have influenced the results of the translocation experiment, these factors might affect the success of naturally deposited eggs and free-living larvae and may thus have contributed to the evolution and maintenance of the differences between subpopulations.

Another mechanism that may have shaped experimental results and driven local adaptation is susceptibility to disease [61]. Host species are exposed to a multitude of parasites and pathogens in their natural habitat [62], and fish ecotypes are often confronted with contrasting parasite communities [63, 64]. Future studies should investigate whether differences in pathogen and parasite faunas between these spawning habitats have played a role in shaping the patterns reported in the present study.

### Natal homing and spawning migration may reinforce population divergence

Differences among populations may evolve as a result of divergent selection and local adaptation [65, 66], random processes such as drift [65], or as a consequence of matching habitat choice [67], or even spatial sorting [68]. In our study system, natal homing and spawning migration behaviour may have promoted population differentiation by constraining gene flow [34, 37, 43]. Legget [69] suggests that homing to natal spawning areas can facilitate evolution of local adaptations through reproductive isolation, and ensure that populations continue to occupy habitats compatible with their life-history. This fits well with our present results. *Esox lucius* seem to choose the same spawning habitat from which they originate and this may have led to reproductive isolation, reinforced local adaptations of juvenile life stages, and possibly resulted in different reproductive allocation strategies. With increasing degree of adaptations to local spawning habitats, selection for natal homing and the differential allocation strategies documented in this study should become stronger.

## Conclusions

Our findings provide support of evolutionary divergence among sympatric subpopulations in which individuals only are physically separated for a short period during reproduction and early development. Results further uncovered differences in reproductive effort and egg size between females from the two subpopulations. These differences in number and size of eggs might reflect counter-adaptations to differences between spawning habitats, such as sedimentation on eggs, or in the intensity of competition and cannibalism. This study emphasizes the significance of investigating life-history traits across different life stages when studying divergence among populations, which makes it important from a pure scientific perspective. Our results are also important in the light of management and conservation of biodiversity. If local adaptations of sympatric subpopulations are overlooked or not taken into consideration, supplementation and reintroduction efforts might have consequences that are detrimental for population fitness.

## References

[1] Endler JA. Geographic variation, speciation and clines. Princeton University Press, Princeton, NJ 1977. 409931

[2] Hereford J. A Quantitative Survey of Local Adaptation and Fitness Trade-Offs. Am Nat. 2009;173(5):579–88. 10.1086/597611 WOS:000264812800004. 19272016

[3] Richter-Boix A, Quintela M, Kierczak M, Franch M, Laurila A. Fine-grained adaptive divergence in an amphibian: genetic basis of phenotypic divergence and the role of nonrandom gene flow in restricting effective migration among wetlands. Mol Ecol. 2013;22(5):1322–40. 10.1111/mec.12181 23294180

[4] Fraser DJ, Bernatchez L. Adaptive migratory divergence among sympatric brook charr populations. Evolution. 2005;59(3):611–24. 15856703 10.1554/04-346

[5] Palumbi SR. GENETIC-DIVERGENCE, REPRODUCTIVE ISOLATION, AND MARINE SPECIATION. Annu Rev Ecol Syst. 1994;25:547–72. 10.1146/annurev.ecolsys.25.1.547 WOS:A1994PU88300022.

[6] Williams GC. Adaptation and Natural Selection. Princeton University Press, Princeton 1966.

[7] Jensen LF, Hansen MM, Pertoldi C, Holdensgaard G, Mensberg KLD, Loeschcke V. Local adaptation in brown trout early life-history traits: implications for climate change adaptability. P Roy Soc B-Biol Sci. 2008;275(1653):2859–68. 10.1098/rspb.2008.0870 WOS:000260611200010. PMC2605839 18755673

[8] Roff DA. The evolution of life histories: theory and analysis. New York: Chapman & Hall Inc.; 1992.

[9] Reznick D, Endler JA. The impact of predation on life history evolution in trinidadian guppies *Poecilia reticulata*. Evolution. 1982;36:160–77. 28581096 10.1111/j.1558-5646.1982.tb05021.x

[10] Johnston TA, Leggett WC. Maternal and environmental gradients in the egg size of an iteroparous fish. Ecology. 2002;83(7):1777–91. 10.2307/3071764 WOS:000176718100001.

[11] Leggett WC, Carscadden JE. Latitudinal variation in reproductive characteristics of American shad (*Alosa-Sapidissima*)—evidence for population specific life history strategies in fish J Fish Res Board Can. 1978;35(11):1469–78 WOS:A1978FW88600010.

[12] Forsman A, Shine R. Parallel geographic variation in body shape and reproductive life history within the Australian scincid lizard Lampropholis delicata. Funct Ecol. 1995;9(6):818–28. ISI:A1995TU96300003.

[13] Smith CC, Fretwell SD. The optimal balance between size and number of offspring. Am Nat. 1974;108:499–506.

[14] Jonsson N, Jonsson B. Trade-off between egg mass and egg number in brown trout. J Fish Biol. 1999;55(4):767–83. WOS:000083040600007.

[15] Stearns SC. Life history evolution: successes, limitations, and prospects. Naturwissenschaften. 2000;87(11):476–86. 10.1007/s001140050763 WOS:000165890100002. 11151666

[16] Leimar O. Environmental and genetic cues in the evolution of phenotypic plasticity. Evol Ecol. 2009;23:125–35.

[17] Forsman A. Rethinking phenotypic plasticity and its consequences for individuals, populations and species. Heredity. 2015;115:276–84. 10.1038/hdy.2014.92 25293873 PMC4815454

[18] Bradshaw AD. Evolutionary significance of phenotypic plasticity in plants. Adv Genet. 1965;13:115–55.

[19] Roff DA. Evolutionary quantitative genetics. New York: Chapman & Hall; 1997.

[20] Pigliucci M. Phenotypic Plasticity Beyond Nature and Nurture. Baltimore, The John Hopkins University Press 2001.

[21] West-Eberhard MJ. Developmental plasticity and evolution. Oxford: Oxford University Press; 2003.

[22] Merilä J, Hendry AP. Climate change, adaptation, and phenotypic plasticity: the problem and the evidence. Evol Appl. 2014;7(1):1–14. 10.1111/eva.12137 24454544 PMC3894893

[23] Via S, Lande R. Genotype-environment interaction and the evolution of phenotypic plasticity. Evolution. 1985;39(3):505–22. 10.2307/2408649 WOS:A1985AJY2600003. 28561964

[24] Pigliucci M. Phenotypic Plasticity Beyond Nature and Nurture. Baltimore: The John Hopkins University Press; 2001 328 p.

[25] Hughes AR, Inouye BD, Johnson MTJ, Underwood N, Vellend M. Ecological consequences of genetic diversity. Ecol Lett. 2008;11:609–23. 10.1111/j.1461-0248.2008.01179.x 18400018

[26] Lucek K, Sivasundar A, Seehausen O. Evidence of Adaptive Evolutionary Divergence during Biological Invasion. Plos One. 2012;7(11). 10.1371/journal.pone.0049377 WOS:000311234000066. PMC3495884 23152900

[27] Wennersten L, Forsman A. Population-level consequences of polymorphism, plasticity and randomized phenotype switching: a review of predictions. Biol Rev. 2012;87(3):756–67. 10.1111/j.1469-185X.2012.00231.x WOS:000305992300015. 22540928

[28] Forsman A, Wennersten L. Inter-individual variation promotes ecological success of populations and species: evidence from experimental and comparative studies. Ecography. 2015;38:10.1111/ecog.01357

[29] Laikre L, Palm S, Ryman N. Genetic population structure of fishes: Implications for coastal zone management. Ambio. 2005;34(2):111–9. 10.1639/0044-7447(2005)034[0111:gpsofi]2.0.co;2 WOS:000228090700008. 15865307

[30] Forsman A. Effects of genotypic and phenotypic variation on establishment are important for conservation, invasion, and infection biology. P Natl Acad Sci USA. 2014;111(1):302–7. 10.1073/pnas.1317745111 WOS:000329350700080. PMC3890895 24367109

[31] Kawecki TJ, Ebert D. Conceptual issues in local adaptation. Ecol Lett. 2004;7(12):1225–41. 10.1111/j.1461-0248.2004.00684.x WOS:000225078000013.

[32] Garcia de Leaniz C, Fleming IA, Einum S, Verspoor E, Jordan WC, Consuegra S, et al A critical review of adaptive genetic variation in Atlantic salmon: implications for conservation. Biol Rev. 2007;82(2):173–211. 10.1111/j.1469-185X.2006.00004.x WOS:000245603300001. 17437557

[33] Forsman A, Tibblin P, Berggren H, Nordahl O, Koch-Schmidt P, Larsson P. Pike Esox lucius as an emerging model organism for studies in ecology and evolutionary biology: a review. J Fish Biol. 2015;87(2):472–9. 10.1111/jfb.12712 WOS:000359421400017. 26077107 PMC4744780

[34] Tibblin P, Forsman A, Koch-Schmidt P, Nordahl O, Johannessen P, Nilsson J, et al Evolutionary Divergence of Adult Body Size and Juvenile Growth in Sympatric Subpopulations of a Top Predator in Aquatic Ecosystems. Am Nat. 2015;186(1):98–110. 10.1086/681597 WOS:000356632700011. 26098342

[35] Craig JF, editor. Pike—biology and exploitation. London: Chapman & Hall; 1996.

[36] Craig JF. A short review of pike ecology. Hydrobiologia. 2008;601:5–16. 10.1007/s10750-007-9262-3 WOS:000253200500002.

[37] Larsson P, Tibblin P, Koch-Schmidt P, Engstedt O, Nilsson J, Nordahl O, et al Ecology, evolution, and management strategies of northern pike populations in the Baltic Sea. Ambio. 2015;44 Suppl 3:451–61. 10.1007/s13280-015-0664-6 MEDLINE:26022327. 26022327 PMC4447694

[38] Engstedt O, Stenroth P, Larsson P, Ljunggren L, Elfman M. Assessment of natal origin of pike (Esox lucius) in the Baltic Sea using Sr:Ca in otoliths. Environ Biol Fish. 2010;89(3–4):547–55. 10.1007/s10641-010-9686-x WOS:000284976500030.

[39] Nilsson J, Engstedt O, Larsson P. Wetlands for northern pike (*Esox lucius L*.) recruitment in the Baltic Sea. Hydrobiologia. 2014;721(1):145–54. 10.1007/s10750-013-1656-9 WOS:000327129300014.

[40] Tibblin P, Berggren H, Nordahl O, Larsson P, Forsman A. Vertebral counts count: Causes and consequences of intra-specific variation in vertebral number. Scientific Reports. *under review*. 10.1038/srep26372 PMC4876516 27210072

[41] Balon EK. REPRODUCTIVE GUILDS OF FISHES—PROPOSAL AND DEFINITION. J Fish Res Board Can. 1975;32(6):821–64. WOS:A1975AC60100016.

[42] Engstedt O, Engkvist R, Larsson P. Elemental fingerprinting in otoliths reveals natal homing of anadromous Baltic Sea pike (*Esox lucius L*.). Ecology of Freshwater Fish. 2014;23:313–21. 10.1111/eff.12082

[43] Tibblin P, Forsman A, Borger T, Larsson P. Causes and consequences of repeatability, flexibility and individual fine-tuning of migratory timing in pike. J Anim Ecol. 2016;85(1):136–45. 10.1111/1365-2656.12439 26412457

[44] Raat AJP. Synopsis of biological data on the northern pike. Rome, Italy: 1988.

[45] Murry BA, Farrell JM, Schulz KL, Teece MA. The effect of egg size and nutrient content on larval performance: implications to protracted spawning in northern pike (*Esox lucius Linnaeus*). Hydrobiologia. 2008;601:71–82. 10.1007/s10750-007-9267-y WOS:000253200500007.

[46] Bolker BM, Brooks ME, Clark CJ, Geange SW, Poulsen JR, Stevens MHH, et al Generalized linear mixed models: a practical guide for ecology and evolution. Trends Ecol Evol. 2009;24(3):127–35. 10.1016/j.tree.2008.10.008 19185386

[47] Rossiter MC. Incidence and consequences of inherited environmental effects. Annu Rev Ecol Syst. 1996;27:451–76. 10.1146/annurev.ecolsys.27.1.451 WOS:A1996VW79800015.

[48] Heath DD, Fox CW, Heath JW. Maternal effects on offspring size: Variation through early development of chinook salmon. Evolution. 1999;53(5):1605–11. 10.2307/2640906 28565562

[49] Stearns SC, Koella JC. The evolution of phenotypic plasticity in life-history traits—predictions of reaction norms for age and size at maturity. Evolution. 1986;40(5):893–913. 10.2307/2408752 WOS:A1986E339300001. 28556219

[50] Scheiner SM. Genetics and evolution of phenotypic plasticity. Annu Rev Ecol Syst. 1993;24:35–68. 10.1146/annurev.es.24.110193.000343 WOS:A1993MJ37100002.

[51] Roff DA. The evolution of life-history parameters in teleosts. Can J Fish Aquat Sci. 1984;41(6):989–1000. WOS:A1984SY55400015.

[52] Gregersen F, Haugen TO, Larsen ON. Egg size differentiation among sympatric demes of brown trout: possible effects of density-dependent interactions among fry. Ecol Fresw Fish. 2006;15(3):237–46. 10.1111/j.1600-0633.2006.00129.x WOS:000239621300001.

[53] Hassler TJ. ENVIRONMENTAL INFLUENCES ON EARLY DEVELOPMENT AND YEAR-CLASS STRENGTH OF NORTHERN PIKE IN LAKES OAHE AND SHARPE, SOUTH-DAKOTA. T Am Fish Soc. 1970;99(2):369-& 10.1577/1548-8659(1970)99<369:eioeda>2.0.co;2 WOS:A1970G263900010.

[54] Auld AH, Schubel JR. Effects of suspended sediment on fish eggs and larvae: A laboratory assessment. Est Coast Mar Sci. 1978;6(2):153–64. 10.1016/0302-3524(78)90097-X

[55] Brooks S, Tyler CR, Sumpter JP. Egg quality in fish: what makes a good egg? Reviews in Fish Biology and Fisheries. 7(4):387–416. 10.1023/a:1018400130692

[56] Vandenberghe EP, Gross MR. Natural-selection resulting from female breeding competition in a pacific salmon (coho, *Oncorhynchus kisutch*). Evolution. 1989;43(1):125–40. 10.2307/2409169 WOS:A1989R828900010. 28568484

[57] Van Noordwijk AJ, Dejong G. Acquisition and allocation of resources—their influences on variation in life-history tactics. Am Nat. 1986;128(1):137–42. 10.1086/284547 WOS:A1986D336700013.

[58] Reznick D, Nunney L, Tessier A. Big houses, big cars, superfleas and the costs of reproduction. Trends Ecol Evol. 2000;15(10):421–5. 10.1016/s0169-5347(00)01941-8 WOS:000089443300018. 10998520

[59] Arendt JD, Reznick DN. Evolution of juvenile growth rates in female guppies (*Poecilia reticulata*): predator regime or resource level? Proc R Soc B. 2005;272(1560):333–7. 10.1098/rspb.2004.2899 BIOSIS:PREV200510008055. 15705560 PMC1634969

[60] Taborsky B. The influence of juvenile and adult environments on life-history trajectories. Proc R Soc B. 2006;273(1587):741–50. 10.1098/rspb.2005.3347 BIOSIS:PREV200600297467. 16608695 PMC1560075

[61] Matthews B, Harmon LJ, M'Gonigle L, Marchinko KB, Schaschl H. Sympatric and Allopatric Divergence of MHC Genes in Threespine Stickleback. Plos One. 2010;5(6). 10.1371/journal.pone.0010948 WOS:000278886100002. PMC2886830 20585386

[62] Eizaguirre C, Lenz TL, Kalbe M, Milinski M. Divergent selection on locally adapted major histocompatibility complex immune genes experimentally proven in the field. Ecol Lett. 2012;15(7):723–31. 10.1111/j.1461-0248.2012.01791.x WOS:000305000000011. 22583762 PMC3440595

[63] Kalbe M, Wegner KM, Reusch TBH. Dispersion patterns of parasites in 0+ year three-spined sticklebacks: a cross population comparison. J Fish Biol. 2002;60(6):1529–42. WOS:000178406000014.

[64] Evans ML, Neff BD, Heath DD. MHC-mediated local adaptation in reciprocally translocated Chinook salmon. Conserv Genet. 2010;11(6):2333–42. 10.1007/s10592-010-0119-3 WOS:000283505900021.

[65] Hendry AP. Adaptive divergence and the evolution of reproductive isolation in the wild: an empirical demonstration using introduced sockeye salmon. Genetica. 2001;112:515–34. 10.1023/a:1013367100865 WOS:000173029400031. 11838786

[66] Junge C, Vollestad LA, Barson NJ, Haugen TO, Otero J, Saetre GP, et al Strong gene flow and lack of stable population structure in the face of rapid adaptation to local temperature in a spring-spawning salmonid, the European grayling (*Thymallus thymallus*). Heredity. 2011;106(3):460–71. 10.1038/hdy.2010.160 WOS:000287636000006. 21224882 PMC3131973

[67] Edelaar P, Siepielski AM, Clobert J. Perspective—Matching habitat choice causes directed gene flow: a neglected dimension in evolution and ecology. Evolution. 2008;62:2462–72. 10.1111/j.1558-5646.2008.00459.x 18637835

[68] Berggren H, Tinnert J, Forsman A. Spatial sorting may explain evolutionary dynamics of wing polymorphism in pygmy grasshoppers. J Evolution Biol. 2012;25(10):2126–38. 10.1111/j.1420-9101.2012.02592.x WOS:000308645100018. 22901281

[69] Legget WC. The Ecology of Fish Migrations. Annu Rev Ecol Syst. 1977;8:285–308.

